# 4-(Dimethyl­amino)phenyl ethynyl telluride

**DOI:** 10.1107/S1600536809009404

**Published:** 2009-03-25

**Authors:** Joan Farran, Angel Alvarez-Larena, Joan F. Piniella, Mario V. Capparelli

**Affiliations:** a Unitat de Cristal·lografia, Universitat Autònoma de Barcelona, 08193 Bellaterra, Spain

## Abstract

The title compound, C_10_H_11_NTe, is the first organyl ethynyl telluride, *R*—Te—C C—H, to be structurally characterized. In the L-shaped mol­ecule, the aryl moiety, *viz.* Me_2_NC_6_H_4_Te, is almost perpendicular to the Te—C C—H fragment. The Te—C*sp* ^2^ bond [2.115 (3) Å] is significantly longer than the Te—C*sp* bond [2.041 (4) Å]. The Te—C C group is approximately linear [Te—C—C = 178.5 (4)° and C C = 1.161 (5) Å], while the coordination at the Te atom is angular [C—Te—C = 95.92 (14)°]. In the crystal structure, there are C*sp*—H⋯N hydrogen bonds which are perpendicular to the CNMe_2_ group; the N atom displays some degree of pyramidalization. Centrosymmetrically related pairs of mol­ecules are linked by Te⋯π(ar­yl) inter­actions, with Te⋯*Cg* = 3.683 (4) Å and C*sp*—Te⋯*Cg* = 159.1 (2)° (*Cg* is the centroid of the benzene ring). These inter­actions lead to the formation of zigzag ribbons which run along *c* and are approximately parallel to (110).

## Related literature

For general background, see: Dabdoub *et al.* (1998 ▶); Gillespie & Hargittai (1991 ▶); Kauffmann & Ahlers (1983 ▶); Murai *et al.* (1994 ▶); Petragnani (1994 ▶); Potapov & Trofimov (2005 ▶); Schulz Lang *et al.* (2006 ▶); Yoshimatsu (2005 ▶); Zukerman-Schpector & Haiduc (2001 ▶). For related structures, see: Farran *et al.* (2002 ▶). For details of the synthesis, see: Brandsma (1988 ▶); Petragnani *et al.* (1975 ▶). 
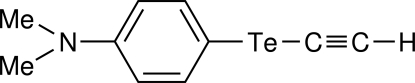


## Experimental

### 

#### Crystal data

C_10_H_11_NTe
*M* *_r_* = 272.80
Triclinic, 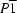

*a* = 7.8857 (7) Å
*b* = 8.3851 (8) Å
*c* = 9.3364 (9) Å
α = 65.788 (2)°
β = 66.922 (1)°
γ = 83.444 (2)°
*V* = 517.18 (8) Å^3^
*Z* = 2
Mo *K*α radiation
μ = 2.82 mm^−1^
*T* = 294 K
0.36 × 0.30 × 0.10 mm

#### Data collection

Bruker SMART APEX diffractometer
Absorption correction: multi-scan (*SADABS*; Bruker, 2001 ▶) *T*_min_ = 0.403, *T*_max_ = 0.754
3574 measured reflections
2401 independent reflections
2080 reflections with *I* > 2σ(*I*)
*R*_int_ = 0.012

#### Refinement

*R*[*F* ^2^ > 2σ(*F* ^2^)] = 0.034
*wR*(*F* ^2^) = 0.087
*S* = 1.04
2401 reflections
110 parameters
H-atom parameters constrained
Δρ_max_ = 0.75 e Å^−3^
Δρ_min_ = −0.35 e Å^−3^

### 

Data collection: *SMART* (Bruker, 2002 ▶); cell refinement: *SAINT* (Bruker, 2001 ▶); data reduction: *SAINT*; program(s) used to solve structure: *SHELXS97* (Sheldrick, 2008 ▶); program(s) used to refine structure: *SHELXL97* (Sheldrick, 2008 ▶); molecular graphics: *ORTEP-3* (Farrugia, 1997 ▶); software used to prepare material for publication: *WinGX* (Farrugia, 1999 ▶) and *PLATON* (Spek, 2009 ▶).

## Supplementary Material

Crystal structure: contains datablocks global, I. DOI: 10.1107/S1600536809009404/bg2241sup1.cif

Structure factors: contains datablocks I. DOI: 10.1107/S1600536809009404/bg2241Isup2.hkl

Additional supplementary materials:  crystallographic information; 3D view; checkCIF report

## Figures and Tables

**Table 1:** Hydrogen-bond geometry (Å, °)

*D*—H⋯*A*	*D*—H	H⋯*A*	*D*⋯*A*	*D*—H⋯*A*
C2—H2⋯N1^i^	0.93	2.48	3.379 (6)	163

Symmetry code: (i) 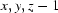.

**Fig. 1.**
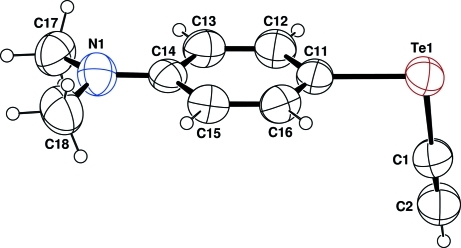
Molecular structure of the title compound showing the atomic numbering. Displacement ellipsoids are drawn at 50% probability level.

**Fig. 2.**
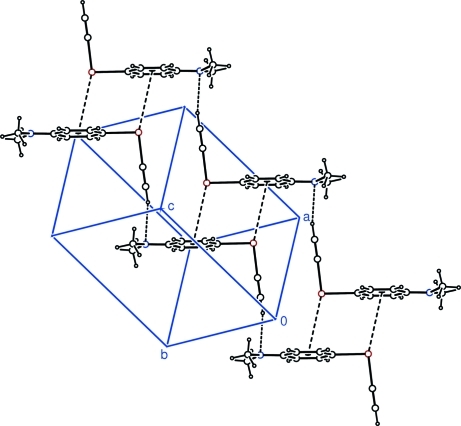
View of the zigzag ribbon generated by the hydrogen bonds and the Te···π(aryl) interactions (shown as dashed lines).

## supplementary crystallographic information

### Comment

Although organotellurium compounds have attracted considerable interest as reagents and intermediates in organic synthesis (Petragnani, 1994), only a limited number of compounds with mono- and ditelluroethyne cores, R—Te—C≡CH and R—Te—C≡C-Te—*R*', have been reported, in spite of the potential reactivity of the acetylene unit towards addition reactions. We recently reported the syntheses and crystal structures of several symmetrical (*R* = *R*') bis(aryltelluro)ethynes, Ar—Te—C≡C-Te—Ar (Farran *et al.*, 2002). On the other hand, only five R—Te—C≡CH derivatives have been prepared so far, with *R* = Me, Et, ^i^Pr, n-Bu and Ph (Kauffmann & Ahlers, 1983; Dabdoub *et al.*, 1998; Potapov & Trofimov, 2005; Yoshimatsu, 2005), and none has been structurally characterized (in addition, molecular orbital calculations for *R* = HC≡C were carried out by Murai *et al.*, 1994). Here we describe the crystal structure of the title compound (*R* = *p*-Me_2_NC_6_H_4_), the first of an organyl ethynyl telluride to be reported.

The structure analysis showed that the crystal contains discrete *L*-shaped molecules of the title compound (Figure 1), in which the aryl moiety, Me_2_NC_6_H_4_Te, is almost perpendicular to the Te—C≡C-H fragment (*cf.* C—Te—C angle, Table 1), but bent *ca* 13° towards the C12—C13 side of the ring (*cf.* C—Te—C—C angles, Table 1), probably to optimize the C—H···N interaction (see below).

As expected, the Te—C(*sp*^2^) bond is significantly longer than the Te—C(*sp*) one. The Te—C≡C moiety is approximately linear, while the coordination at the Te atom is angular, as predicted by the valence-shell electron-pair repulsion (VSEPR) model for an A*X*_2_E_2_ molecule (Gillespie & Hargittai, 1991). The values of these geometric parameters (Table 1) are similar to the ranges observed in several bis(arytelluro)ethynes, Ar—Te—C≡C-Te—Ar (Farran *et al.*, 2002), *viz*. Te—C(*sp*^2^), 2.103 (5)–2.142 (6) Å; Te—C(*sp*), 2.021 (6)–2.058 (6) Å; C≡C, 1.166 (12)–1.203 (11) Å and C—Te—C, 94.2 (3)–97.2 (2)°, which are substantially smaller than the tetrahedral value (109.5°) due to the repulsion of the lone pairs of electrons on the bonded ones.

In the crystal structure the molecules are linked by C(*sp*)—H···N hydrogen bonds (Table 2) which are perpendicular to the CNMe_2_ group. The N atom displays some degree of pyramidalization: it is 0.123 (5) Å out of the plane of the three C atoms, towards the H atom. There are also Te···π(aryl) interactions, similar to those described by Zukerman-Schpector & Haiduc (2001) or Schulz Lang *et al.* (2006) for Te(IV) compounds, in which centrosymmetrically related pairs of molecules are at Te···*Cg* 3.683 (4) Å and C(*sp*)—Te···*Cg* 159.1 (2)° (*Cg* = centroid of the phenyl ring at 1 - *x*, -*y*, 1 - *z*). These interactions lead to the formation of zigzag ribbons, made of pairs of chains, which run along c and are approximately parallel to (110) (Figure 2).

### Experimental

Ethynyl magnesium bromide, HC≡CMgBr, was prepared according to published procedures (Brandsma, 1988). The corresponding diaryl ditelluride, (Me_2_NC_6_H_4_Te)_2_, was synthesized as reported elsewhere (Petragnani *et al.*, 1975). A dark solution of the diaryl ditelluride (2.0 mmol, 0.94 g) in 40 ml of THF was treated dropwise with bromine (2.0 mmol, 0.32 g, 0.10 ml) in 10 ml of benzene, at 0°C, in N_2_ atmosphere, while efficient cooling was applied. The Grignard reagent was then added dropwise. Gradual disappearance of the dark color of the solution was observed until it finally became almost colorless when about 10% excess of the reagent was added. After stirring for 30 min at room temperature, the solution was diluted with 50 ml of low boiling point petroleum ether, treated with aqueous NH_4_Cl and washed with brine. The organic layer was dried over magnesium sulfate and the solvents were evaporated. The residue was purified by flash chromatography (silica gel/hexane). Yield 51%. Crystals suitable for X-ray analysis were obtained by slow evaporation of a chloroform solution. The specimen used for data collection was air-protected with a thin coat of Loctite epoxy adhesive.

### Refinement

Hydrogen atoms were placed in calculated positions using a riding atom model with fixed C—H distances [0.93 Å for C(*sp*) and C(*sp*^2^), 0.96 Å for C(*sp*^3^)] and *U*_iso_ = p *U*_eq_(parent atom) [p = 1.2 for C(*sp*) and C(*sp*^2^), 1.5 for C(*sp*^3^)].

### Crystal data

**Table:**

C_10_H_11_NTe	*Z* = 2
*M**_r_* = 272.80	*F*(000) = 260
Triclinic, *P*1	*D*_x_ = 1.752 Mg m^−^^3^
Hall symbol: -P 1	Mo *K*α radiation, λ = 0.71073 Å
*a* = 7.8857 (7) Å	Cell parameters from 1735 reflections
*b* = 8.3851 (8) Å	θ = 2.7–25.6°
*c* = 9.3364 (9) Å	µ = 2.82 mm^−^^1^
α = 65.788 (2)°	*T* = 294 K
β = 66.922 (1)°	Plate, pale brown
γ = 83.444 (2)°	0.36 × 0.30 × 0.10 mm
*V* = 517.18 (8) Å^3^	

### Data collection

**Table:**

Brruker SMART APEX diffractometer	2401 independent reflections
Radiation source: fine-focus sealed tube	2080 reflections with *I* > 2σ(*I*)
graphite	*R*_int_ = 0.012
Detector resolution: 8.13 pixels mm^-1^	θ_max_ = 28.9°, θ_min_ = 2.6°
φ and ω scans	*h* = −10→10
Absorption correction: multi-scan (*SADABS*; Bruker, 2001)	*k* = −10→10
*T*_min_ = 0.403, *T*_max_ = 0.754	*l* = −10→12
3574 measured reflections	

### Refinement

**Table:**

Refinement on *F*^2^	Primary atom site location: structure-invariant direct methods
Least-squares matrix: full	Secondary atom site location: difference Fourier map
*R*[*F*^2^ > 2σ(*F*^2^)] = 0.034	Hydrogen site location: inferred from neighbouring sites
*wR*(*F*^2^) = 0.087	H-atom parameters constrained
*S* = 1.04	*w* = 1/[σ^2^(*F*_o_^2^) + (0.0487*P*)^2^ + 0.1129*P*] where *P* = (*F*_o_^2^ + 2*F*_c_^2^)/3
2401 reflections	(Δ/σ)_max_ = 0.005
110 parameters	Δρ_max_ = 0.75 e Å^−^^3^
0 restraints	Δρ_min_ = −0.35 e Å^−^^3^

### Special details

**Table:**

Geometry. All e.s.d.'s (except the e.s.d. in the dihedral angle between two l.s. planes) are estimated using the full covariance matrix. The cell e.s.d.'s are taken into account individually in the estimation of e.s.d.'s in distances, angles and torsion angles; correlations between e.s.d.'s in cell parameters are only used when they are defined by crystal symmetry. An approximate (isotropic) treatment of cell e.s.d.'s is used for estimating e.s.d.'s involving l.s. planes.

### Fractional atomic coordinates and isotropic or equivalent isotropic displacement parameters (Å^2^)

**Table:**

	*x*	*y*	*z*	*U*_iso_*/*U*_eq_	
Te1	0.45985 (3)	0.02042 (3)	0.26991 (3)	0.06634 (13)	
C1	0.3522 (5)	0.1483 (5)	0.0886 (5)	0.0612 (8)	
C2	0.2942 (7)	0.2203 (6)	−0.0170 (6)	0.0747 (11)	
H2	0.2478	0.2779	−0.1016	0.090*	
C11	0.3173 (5)	0.1554 (5)	0.4271 (4)	0.0532 (7)	
C12	0.3748 (5)	0.3243 (5)	0.3871 (5)	0.0608 (9)	
H12	0.4778	0.3792	0.2894	0.073*	
C13	0.2819 (5)	0.4118 (5)	0.4899 (5)	0.0595 (8)	
H13	0.3219	0.5256	0.4592	0.071*	
C14	0.1272 (5)	0.3316 (4)	0.6407 (4)	0.0515 (7)	
C15	0.0702 (5)	0.1609 (5)	0.6793 (5)	0.0568 (8)	
H15	−0.0321	0.1042	0.7771	0.068*	
C16	0.1641 (5)	0.0766 (4)	0.5739 (5)	0.0566 (8)	
H16	0.1236	−0.0362	0.6022	0.068*	
N1	0.0368 (5)	0.4146 (5)	0.7474 (4)	0.0639 (8)	
C17	0.0794 (7)	0.5988 (5)	0.6960 (6)	0.0714 (11)	
H171	0.0033	0.6351	0.7852	0.107*	
H172	0.0561	0.6671	0.5949	0.107*	
H173	0.2071	0.6158	0.6739	0.107*	
C18	−0.1341 (7)	0.3391 (7)	0.8888 (6)	0.0834 (13)	
H181	−0.1834	0.4182	0.9439	0.125*	
H182	−0.1121	0.2302	0.9681	0.125*	
H183	−0.2209	0.3184	0.8485	0.125*	

### Atomic displacement parameters (Å^2^)

**Table:**

	*U*^11^	*U*^22^	*U*^33^	*U*^12^	*U*^13^	*U*^23^
Te1	0.0694 (2)	0.0780 (2)	0.06248 (18)	0.02561 (14)	−0.03447 (14)	−0.03496 (14)
C1	0.061 (2)	0.067 (2)	0.060 (2)	0.0095 (17)	−0.0267 (17)	−0.0276 (17)
C2	0.085 (3)	0.081 (3)	0.069 (2)	0.023 (2)	−0.041 (2)	−0.033 (2)
C11	0.0526 (18)	0.061 (2)	0.0527 (18)	0.0111 (15)	−0.0256 (15)	−0.0263 (15)
C12	0.0518 (18)	0.067 (2)	0.056 (2)	−0.0026 (16)	−0.0174 (16)	−0.0194 (17)
C13	0.060 (2)	0.0532 (19)	0.064 (2)	−0.0041 (16)	−0.0241 (17)	−0.0194 (16)
C14	0.0541 (18)	0.0528 (18)	0.0510 (17)	0.0093 (14)	−0.0274 (15)	−0.0186 (14)
C15	0.0558 (19)	0.0539 (19)	0.0522 (18)	−0.0010 (15)	−0.0163 (15)	−0.0163 (15)
C16	0.064 (2)	0.0460 (17)	0.061 (2)	0.0023 (15)	−0.0293 (17)	−0.0174 (15)
N1	0.070 (2)	0.0569 (17)	0.0628 (19)	0.0078 (14)	−0.0228 (16)	−0.0254 (14)
C17	0.088 (3)	0.062 (2)	0.085 (3)	0.019 (2)	−0.048 (2)	−0.039 (2)
C18	0.076 (3)	0.095 (3)	0.073 (3)	0.013 (2)	−0.015 (2)	−0.043 (2)

### Geometric parameters (Å, °)

**Table:**

Te1—C1	2.041 (4)	C15—C16	1.376 (5)
Te1—C11	2.115 (3)	C15—H15	0.9300
C1—C2	1.161 (5)	C16—H16	0.9300
C2—H2	0.9300	N1—C18	1.440 (6)
C11—C16	1.384 (5)	N1—C17	1.454 (6)
C11—C12	1.390 (5)	C17—H171	0.9600
C12—C13	1.378 (5)	C17—H172	0.9600
C12—H12	0.9300	C17—H173	0.9600
C13—C14	1.410 (5)	C18—H181	0.9600
C13—H13	0.9300	C18—H182	0.9600
C14—N1	1.372 (5)	C18—H183	0.9600
C14—C15	1.407 (5)		
			
C1—Te1—C11	95.92 (14)	C15—C16—C11	121.8 (3)
C2—C1—Te1	178.5 (4)	C15—C16—H16	119.1
C1—C2—H2	180.0	C11—C16—H16	119.1
C16—C11—C12	118.1 (3)	C14—N1—C18	120.5 (3)
C16—C11—Te1	120.6 (3)	C14—N1—C17	120.7 (4)
C12—C11—Te1	121.3 (3)	C18—N1—C17	116.6 (4)
C13—C12—C11	121.2 (3)	N1—C17—H171	109.5
C13—C12—H12	119.4	N1—C17—H172	109.5
C11—C12—H12	119.4	H171—C17—H172	109.5
C12—C13—C14	121.0 (3)	N1—C17—H173	109.5
C12—C13—H13	119.5	H171—C17—H173	109.5
C14—C13—H13	119.5	H172—C17—H173	109.5
N1—C14—C15	121.0 (3)	N1—C18—H181	109.5
N1—C14—C13	121.8 (3)	N1—C18—H182	109.5
C15—C14—C13	117.2 (3)	H181—C18—H182	109.5
C16—C15—C14	120.8 (3)	N1—C18—H183	109.5
C16—C15—H15	119.6	H181—C18—H183	109.5
C14—C15—H15	119.6	H182—C18—H183	109.5
			
C1—Te1—C11—C16	102.7 (3)	C13—C14—C15—C16	0.7 (5)
C1—Te1—C11—C12	−77.8 (3)	C14—C15—C16—C11	0.1 (6)
C16—C11—C12—C13	−0.4 (6)	C12—C11—C16—C15	−0.3 (5)
Te1—C11—C12—C13	−179.9 (3)	Te1—C11—C16—C15	179.3 (3)
C11—C12—C13—C14	1.2 (6)	C15—C14—N1—C18	−8.9 (6)
C12—C13—C14—N1	177.8 (4)	C13—C14—N1—C18	172.1 (4)
C12—C13—C14—C15	−1.3 (5)	C15—C14—N1—C17	−171.4 (4)
N1—C14—C15—C16	−178.4 (3)	C13—C14—N1—C17	9.6 (6)

### Hydrogen-bond geometry (Å, °)

**Table:**

*D*—H···*A*	*D*—H	H···*A*	*D*···*A*	*D*—H···*A*
C2—H2···N1^i^	0.93	2.48	3.379 (6)	163

Symmetry codes: (i) *x*, *y*, *z*−1.
